# New Appliances and Things Medical

**Published:** 1899-11-25

**Authors:** 


### NEW APPLIANCES AND THINGS MEDICAL.

**[We shall be glad to receive, at our Office, 28 & 29, Southampton Street, Strand, London, W.C., from the manufacturers, specimens of all new preparations and appliances which may be brought out from time to time.]**

### PHOSFERINE.

(Ashton and Parsons, 17, Farringdon Road, London, E.C.)

This is a concentrated tonic of good qualities; it apparently has no disturbing influence on digestion. In cases of neurasthenia and nervous debility it should find its most useful province. The dose is from five to eight drops taken in a small quantity of water; it is by no means disagreeable.

### MANHU DIABETIC FOODS.

(Irving, Son, and Jones, Liverpool, and 88, Charing Cross Road, London, W.C.)

The list of Manhu diabetic foods includes flour, biscuits, flaked wheat, flaked barley, and prepared barley. They are foods which are well tolerated in the diabetic state, and are particularly rich in proteid substances. We recommend them to the notice of our readers.

